# Asperuloside Improves Obesity and Type 2 Diabetes through Modulation of Gut Microbiota and Metabolic Signaling

**DOI:** 10.1016/j.isci.2020.101522

**Published:** 2020-09-02

**Authors:** Anna Nakamura, Yoko Yokoyama, Kazuki Tanaka, Giorgia Benegiamo, Akiyoshi Hirayama, Qi Zhu, Naho Kitamura, Taichi Sugizaki, Kohkichi Morimoto, Hiroshi Itoh, Shinji Fukuda, Johan Auwerx, Kazuo Tsubota, Mitsuhiro Watanabe

**Affiliations:** 1 Systems Biology Program, Graduate School of Media and Governance, Keio University, Fujisawa, Kanagawa 252-0882, Japan; 2 Health Science Laboratory, Keio Research Institute at SFC, Fujisawa, Kanagawa 252-0882, Japan; 3 Institute for Advanced Biosciences, Keio University, Tsuruoka, Yamagata 997-0052, Japan; 4 Intestinal Microbiota Project, Kanagawa Institute of Industrial Science and Technology, Kawasaki, Kanagawa 210-0821, Japan; 5 Laboratory of Integrative and Systems Physiology, École Polytechnique Fédérale de Lausanne (EPFL), Lausanne 1015, Switzerland; 6 Department of Environment and Information Studies, Keio University, Fujisawa, Kanagawa 252-0882, Japan; 7 Department of Internal Medicine, Keio University School of Medicine, Shinjuku, Tokyo 160-8582, Japan; 8 Transborder Medical Research Center, University of Tsukuba, Tsukuba, Ibaraki 305-8575, Japan; 9 Department of Ophthalmology, Keio University School of Medicine, Shinjuku, Tokyo 160-8582, Japan

**Keywords:** Human Metabolism, Microbiome

## Abstract

Asperuloside (ASP) is an iridoid glycoside that is extracted from *Eucommia* leaves. *Eucommia* is used in traditional Chinese medicine and has a long history of benefits on health and longevity. Here, we investigated the impact of ASP on obesity-related metabolic disorders and show that ASP reduces body weight gain, glucose intolerance, and insulin resistance effectively in mice fed with a high-fat diet (HFD). Intestinal dysbiosis is closely linked with metabolic disorders. Our data indicate that ASP achieves these benefits on metabolic homeostasis by reversing HFD-induced gut dysbiosis and by changing gut-derived secondary metabolites and metabolic signaling. Our results indicate that ASP may be used to regulate gut microbiota for the treatment of obesity and type 2 diabetes.

## Highlights

• Asperuloside (ASP) treatment improved obesity-related metabolic dysfunction in mice
ASP administration changed intestinal metabolite via modulation of gut microbiota
ASP administration increased *Parabacteroides* and *Akkermansia* in intestine
Gut microbiota-derived metabolite improves adipose tissue and intestinal dysfunction

## Graphical Abstract

**Figure:**
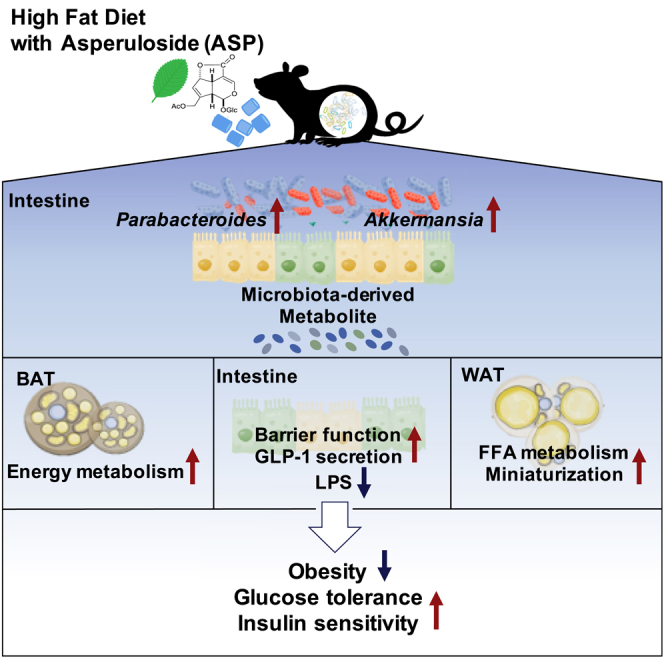

## Introduction

Obesity has recently become epidemic, with one in three people being obese or overweight worldwide, and as such poses a major public health problem (Hoyt et al., 2014). Obesity has been linked to a variety of chronic diseases often within the context of the metabolic syndrome, a cluster of symptoms that include insulin resistance, type 2 diabetes, cardiovascular diseases, fatty liver, and even liver cancer (Osborn and Olefsky, 2012). According to recent data, extreme obesity could shorten the average life expectancy by about 14 years (Kitahara et al., 2014) and substances that prevent obesity could hence lead to the extension of a healthy lifespan.

Obesity results from the interaction of many genetic, metabolic, and environmental factors. In recent years, knowledge about the gut microbiota in health has grown tremendously (Cani and Delzenne, 2009). Intestinal bacteria contribute to the pathogenesis of obesity through a wide range of mechanisms that include, but are not restricted to, the promotion of energy absorption from diet, alterations in the production of intestinal hormones, and the induction of insulin resistance by obesity-induced inflammation (Bäckhed et al., 2007; Kimura et al., 2013; Ridaura et al., 2013). Antibiotics and prebiotics are being evaluated for the management of obesity and related metabolic disorders. Administration of antibiotics improves impaired glucose tolerance, and prebiotics reduce the influx of the endotoxin, lipopolysaccharide (LPS), by strengthening the barrier function of the intestinal tract. Antibiotics and prebiotics have also been reported to suppress and control obesity-induced inflammation (Cani et al., 2008, 2009). Also, recent studies revealed that some bacteria can change metabolic signaling pathways via their secondary metabolites. For example, in patients with type 2 diabetes, the intestinal bacteria composition and metabolites are different from those of healthy subjects (Sato et al., 2014). In addition, the presence of *Akkermansia* has been proposed to maintain gut health and glucose homeostasis, and recently, *Akkermansia* abundance has been linked with increased level of nicotinamide (Blacher et al., 2019). Also, *Parabacteroides* have been shown to attenuate obesity and metabolic dysfunctions via the production of succinate and secondary bile acids (Wang et al., 2019). Based on all these evidences, it is clear that change in the gut microbiota and their secondary metabolites are closely linked to the development of metabolic disease.

Asperuloside (ASP) is an iridoid glycoside derived from the leaves of the *Eucommia* tree (*Eucommia ulmoides Oliv*.). Its bark (Cortex Eucommiae) has been traditionally used in Chinese medicine for its analeptic, analgesic, sedative, antihypertensive, and diuretic properties. In recent years, *Eucommia* leaves have been shown to curb obesity (Hosoo et al., 2015), an effect that was linked to the main active compound, asperuloside (ASP) (Hirata et al., 2011). A previous study showed that administration of ASP reduced body weight gain by reducing visceral fat mass (Hirata et al., 2011). However, the underlying mechanism of how ASP affects weight gain and influences other obesity-related disorders remains unknown. Several compounds included in Chinese medicine are metabolized by gut bacteria into active intermediates (Lee et al., 2012). Here we report that ASP changes the gut microbiota composition and the production of secondary metabolites, affects whole-body signaling, and thereby curbs obesity and its related metabolic dysfunctions.

## Results

### ASP Prevents High-Fat-Diet-Induced Obesity

**Figure 1:**
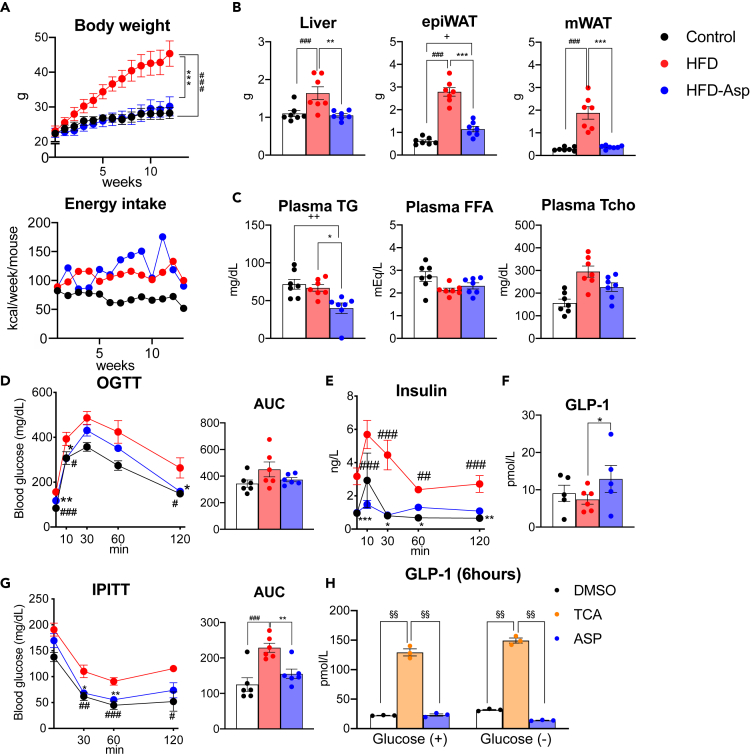
ASP Prevents High-Fat Diet-Induced Obesity and Improves Glucose Tolerance and Insulin Resistance (A–G) Male C57BL/6J mice aged 5 weeks were fed with a control diet (10% kcal fat; Control), a high-fat diet (60% kcal fat; HFD), or a high-fat diet supplemented with 0.25% w/w asperuloside (HFD-Asp) for 12 weeks (n = 5-7 for each group). (A) Body weight evolution and food intake of C57BL/6J mice in the different diet groups. Energy intake was measured as the average food intake per mouse in a week. (B) Comparison of liver, mWAT, and epiWAT tissue weights after 12 weeks of treatment with different diets. (C) Plasma total cholesterol (Tcho), triglyceride (TG), and free fatty acid (FFA) levels after 12 weeks of ASP supplementation. (D) Serum glucose levels and AUC during OGTT. (E) Plasma insulin concentration during OGTT. (F) Plasma active GLP-1 levels 10 min after administration of glucose during the OGTT. (G) The reduction of serum glucose levels and AUC during IPITT. (H) GLP-1 concentration secreted from NCI-H716 cell line cultured for 6 h. (A–G) Results are expressed as mean ± SEM (n = 5–7 mice for each group). ∗p < 0.05, ∗∗p < 0.01, ∗∗∗p < 0.001 HFD versus HFD-Asp, #p < 0.05, ##p < 0.01, ###p < 0.001 HFD versus Control, +p < 0.05, ++p < 0.01, +++p < 0.001 Control versus HFD-Asp. Statistical analysis with one-way ANOVA followed by Tukey's multiple comparison test or Dunnett's multiple comparison test. (H) Results are expressed as mean ± SEM. ^§^p < 0.05, ^§§^ <0.01, versus TCA treatment. Statistical analysis with one-way ANOVA followed by Dunnett's multiple comparison test. See also Figure S1.

First, we evaluated the metabolic phenotype of male mice fed normal chow (control), a high-fat diet (HFD), or a high-fat diet supplemented with ASP (HFD-Asp) for 12 weeks starting at the age of 5 weeks. When compared with animals fed chow, high-fat-fed animals gained significantly more weight. However, mice fed a HFD with ASP were resistant to body weight gain and their body weight remained similar to the control group (Figure 1A). There was no significant difference between the energy intakes of HFD- and ASP-treated mice (Figure 1A). In addition, liver, epididymal white adipose tissue (epiWAT), and mesenteric WAT (mWAT) mass were all decreased in ASP-treated mice (Figure 1B). Furthermore, serum triglycerides (TG) were significantly lower and total cholesterol levels tended to decrease in ASP-treated animals, whereas free fatty acid levels were similar in the ASP and HFD treatment groups (Figure 1C). These results suggest that ASP reduces body weight gain and fat accumulation, as well as serum lipid parameters induced by HFD.

### ASP Improves Glucose Homeostasis and Insulin Resistance

Glucose metabolism is often abnormal in obese patients. Given the robust impact of ASP on body weight gain, we next investigated the effect of ASP on glucose metabolism. The administration of ASP improves glucose clearance during an oral glucose tolerance test (OGTT) (Figure 1D). The lower plasma insulin levels during OGTT indicated that increased insulin sensitivity led to improved glucose metabolism in ASP-treated mice (Figure 1E). Interestingly, the levels of active GLP-1, an incretin secreted from the gut L-cells upon feeding and that induces insulin secretion, was increased in ASP-treated mice (Figure 1F). Furthermore, intraperitoneal insulin tolerance test (IPITT) showed that mice fed HFD became insulin resistant, whereas ASP significantly improved insulin sensitivity (Figure 1G). Taken together, ASP improves glucose tolerance and insulin sensitivity and increases GLP-1 levels. We next tested whether ASP may have a direct effect to stimulate GLP-1 release from intestinal L-cells. We therefore incubated NCI-H716 cells, which secrete GLP-1 (Reimer et al., 2001), with ASP for 30 min and 6 h. As positive control we used the bile acid taurocholic acid (TCA). TCA stimulated GLP-1 secretion both after 30-min and 6-h exposures, whereas we did not observe an increase in GLP-1 concentration when cells were treated with ASP (Figures 1H and S1). This result indicated that the observed effect of ASP on GLP-1 secretion *in vivo* is indirect.

### ASP Reverses High-Fat-Diet-Induced Gut Dysbiosis

**Figure 2:**
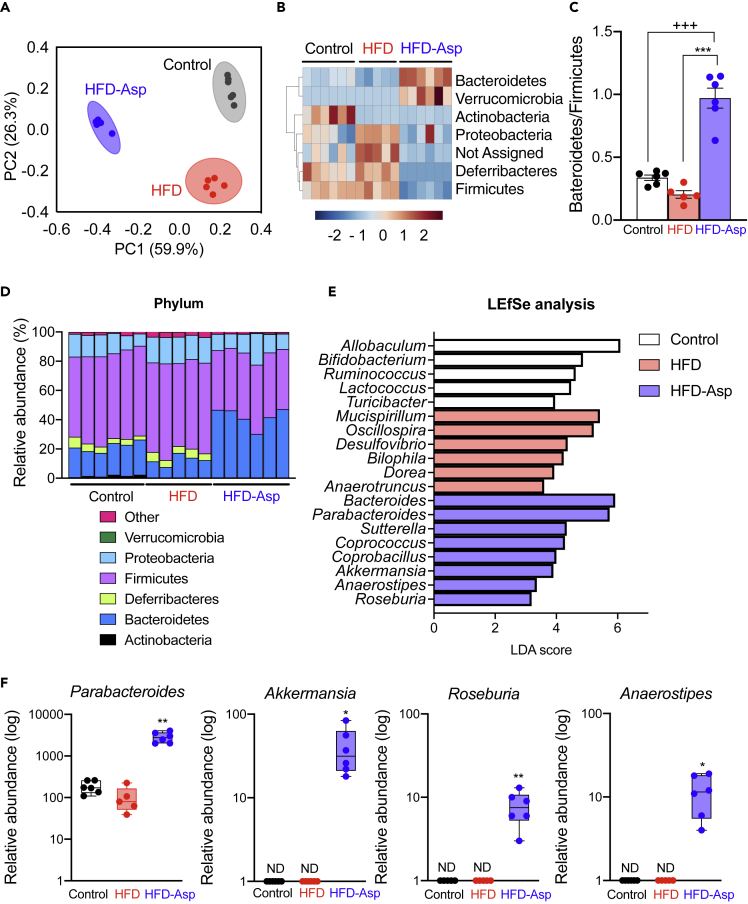
ASP Modulates the Composition of Gut Microbiota Community (A–F) Analysis of the 16S rRNA gene sequences of cecal contents of the mice used in Figure 1. (A) Principal coordinates analysis of the 16S rRNA data. (B) Heatmap of the microbiota components at the class level. (C) The ratio of Bacteroidetes/Firmicutes. (D) Phylum-level taxonomic distributions of the microbial communities in cecal contents of mice fed with the three different diets. (E) Linear discriminant analysis effect size (LEfSe) analysis. (F) Relative abundance of four prototypical bacteria at the genus level. Results are expressed as mean ± SEM (n = 5–6 mice for each group). ∗p < 0.05, ∗∗p < 0.01, ∗∗∗p < 0.001 HFD versus HFD-Asp; #p < 0.05, ##p < 0.01, ###p < 0.001 HFD versus Control; +p < 0.05, ++p < 0.01, +++p < 0.001 Control versus HFD-Asp. See also Figure S2.

Given the importance of the gut microbiome in metabolic homeostasis (Cani and Delzenne, 2009), we next performed bacterial 16S rRNA gene sequencing of cecal samples. First, we evaluated the groups fed with control, HFD, and HFD-ASP diets for changes in the microbiome at the phylum level. UniFrac-based principal coordinates analysis revealed a distinct clustering of the microbiota composition for each treatment group (Figure 2A). Hierarchical clustering at the phylum level showed that the microbial communities in the cecum of the ASP-fed mice were significantly different from those of the control and HFD-fed mice; ASP treatment increased Bacteroidetes and Verrucomicrobia (Figure 2B). Also, the ratio of Bacteroidetes/Firmicutes was increased in the HFD-Asp group (Figure 2C). To access specific changes in microbiota, we analyzed the relative abundance of the predominant taxa identified from sequencing in the three diet groups. Phylum-level changes further confirmed a higher level of Bacteroidetes to Firmicutes in mice that received ASP supplementation (Figure 2D). In genus-level analysis, ASP increased *Bacteroides* and *Parabacteroides* (Figure S2A).

### ASP Increases Beneficial Gut Bacteria in an HFD-Fed Mouse Model

We next investigated, which specific bacteria at the genus level were increased by ASP supplementation. Community structure analysis using correlation analysis at the genus level in HFD versus HFD-Asp group showed that ASP supplementation correlated positively with *Akkermansia*, *Parabacteroides, Bacteroides, Sutterella, Anaerostipes, Roseburia,* and *Coprobacillus* abundance (Figure S2B). Also, linear discriminant analysis effect size analysis shows a similar pattern as that seen in the correlation analysis (Figure 2E). We next measured the relative abundance of four bacteria, i.e., *Akkermansia*, *Roseburia, Anaerostipes,* and *Parabacteroides*; ASP increases the actual abundance of all four bacteria (Figure 2F). *Akkermansia* has been reported to strengthen intestinal tight junctions and control the inflow of LPS into the blood (Everard et al., 2013). *Roseburia* and *Anaerostipes* produce short-chain fatty acids (SCFAs) via metabolization of carbohydrate (Basson et al., 2016), and *Parabacteroides* are known to improve glucose homeostasis by producing succinate (Wang et al., 2019). These results show that ASP changes the microbial community and increases the number of bacteria with a positive health impact.

### ASP Alters Whole-Body Metabolism by Changing Cecal Metabolite Levels

**Figure 3:**
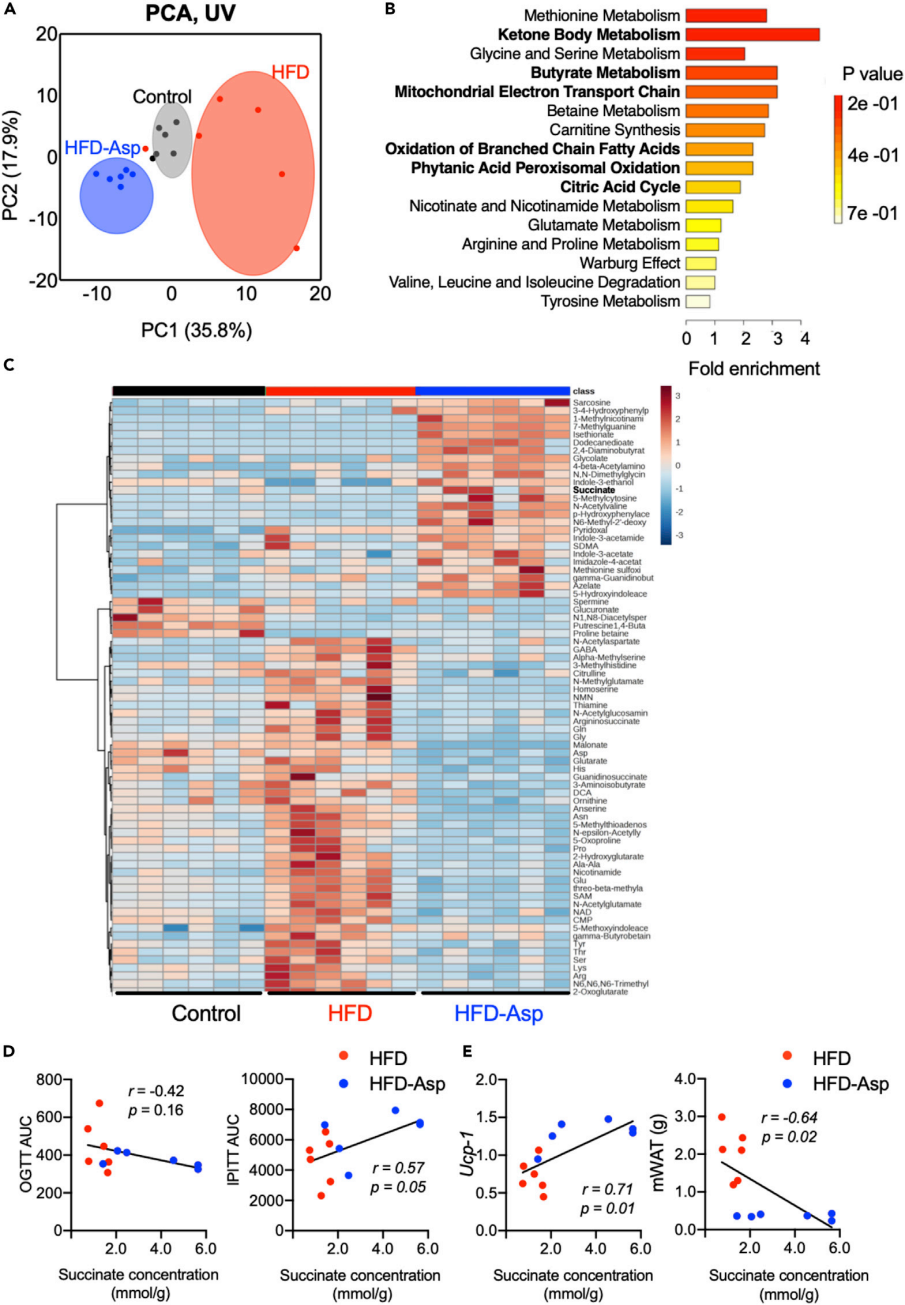
ASP Remodeling of the Cecal Metabolite Profile and Increase in Succinate Concentration (A–E) Cecal metabolome analysis on the mice used in Figure 1. (A) Principal-component analysis (PCA) of the cecal metabolome profiles normalized by unit valiance (UV). (B) Enrichment analysis of cecal metabolites that were changed by more than 2-fold between HFD after ASP supplementation. Bold character shows related to lipid metabolism. (C) Heatmap showing the concentrations of the top 75 metabolites that were quantified. (D) Correlation analysis between succinate concentration and glucose tolerance and insulin sensitivity. (E) Correlation analysis between succinate concentration and *Ucp-1* expression in brown adipose tissue (BAT) and mesenteric white adipose tissue (mWAT) weight.

Given the important changes in the intestinal microbiome analysis, we next measured changes in the metabolome induced by ASP in the cecal content using Capillary electrophoresis with electrospray ionization time-of-flight mass spectrometry (CE-TOFMS). Principal-component analysis of the cecal metabolites showed a clear separation among three diets in the first component (Figure 3A). In mice receiving ASP in the diet the most changed cecal metabolites were related to the metabolism of ketone bodies, butyrate, and other fatty acids (Figure 3B). These results indicate that ASP may mainly affect lipid metabolism. The hierarchical clustering of detected features showed qualitative differences between the HFD and HFD-Asp groups (Figure 3C). In HFD, the levels of glycine, L-glutamic acid, and other amino acid-derived compounds increased. In HFD-Asp, the levels of sarcosine and dimethylglycine (involved in methionine metabolism) and succinate (a metabolite from the TCA cycle, ketone body metabolism, and other lipid metabolism pathway) increased (Figure 3C). Considering the result from the enrichment analysis (Figure 3B), we focused on the lipid metabolite succinate. Succinate, which is detected in HFD-Asp group, is the precursor of SCFAs. In addition, succinate is produced by gut microbiota *Parabacteroides*, which have increased significantly in HFD-Asp mice. It is known to improve glucose intolerance and insulin resistance (De Vadder et al., 2016). In our study, succinate was negatively correlated with the area under the curve (AUC) during OGTT and was positively correlated with the drop rate in glucose during IPITT (AUC below baseline), which are indicators of glucose intolerance and insulin sensitivity, respectively (Figure 3D). From the result of enrichment analysis, we focused on the relationship between adipocyte metabolism and succinate. Recent study reported that the accumulation of succinate activates brown adipose tissue (BAT) thermogenesis (Mills et al., 2018), we also confirmed a positive correlation between *Ucp-1* expression which high expression in BAT and succinate concentration in our study (Figure 3E). Furthermore, there are negative correlations with the weight of each white adipose tissue (WAT) depot and succinate levels (Figures 3E and S3B).

### ASP Improves Adipose Tissue Metabolism

**Figure 4:**
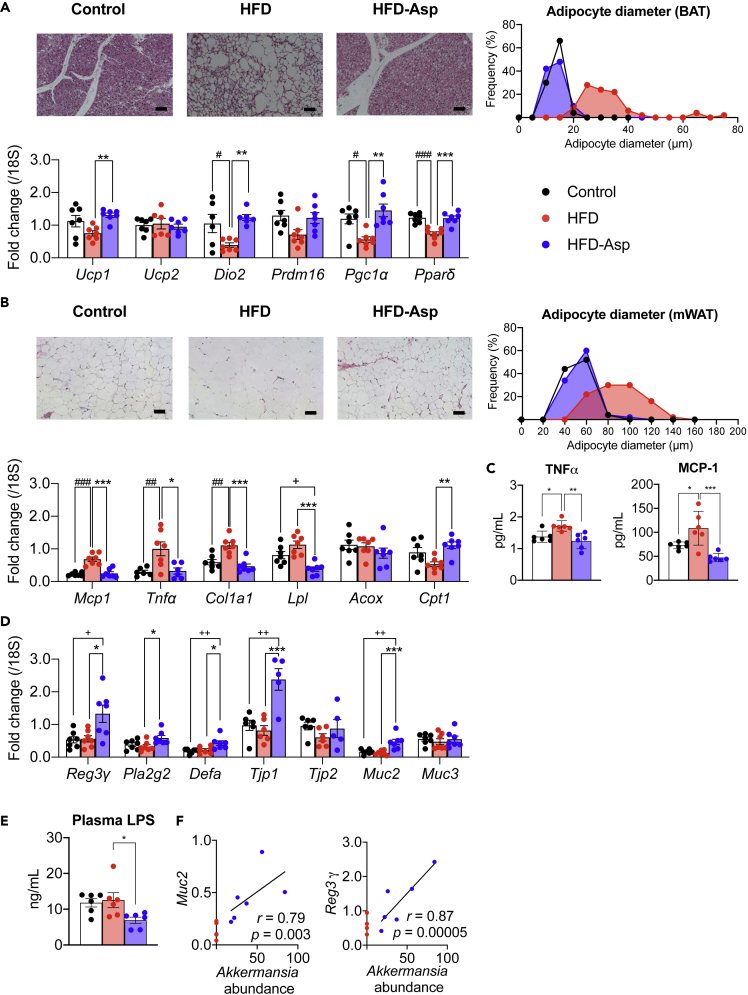
Impact of ASP on Several Organs (A–E) Histological analysis, LPS measurement, and qPCR analysis on the mice used in Figure 1. (A) Histological analysis and expression of mRNA levels of selected genes; qPCR analysis in the brown adipocyte. Brown adipocyte morphology was assessed by H&E staining (scale bar, 50 μm). (B) Histological analysis and expression of mRNA levels of selected genes; qPCR analysis in the mesenteric white adipose tissue (mWAT). Morphology of mWAT was assessed by H&E staining (scale bar, 50 μm). (C) Plasma level of TNFα and MCP-1 analysis measured by ELISA. (D) qPCR analysis in ileum. (E) Plasma LPS concentration. (F) Correlation analysis between *Muc2* and *Reg3*γ expression and *Akkermansia* abundance. y axis: expression of *Muc2* and *Reg3*γ*;* x axis: *Akkermansia* abundance of cecal contents. The intestinal portions are collected from a section 3 cm above the cecum. Results are expressed as mean ± SEM (n = 5–7 mice for each group). ∗p < 0.05, ∗∗p < 0.01, ∗∗∗p < 0.001 HFD versus HFD-Asp; #p < 0.05, ##p < 0.01, ###p < 0.001 HFD versus Control; +p < 0.05, ++p < 0.01, +++p < 0.001 Control versus HFD-Asp. Statistical analysis with one-way ANOVA followed Tukey's multiple comparison test. See also Figure S3 and Table S1.

Obesity is typified by TG accumulation in adipocytes, which become enlarged and hypertrophic. Bloated fat cells secrete tumor necrosis factor alpha (TNF-α) and resistin, which are mediators of insulin resistance (Hotamisligil, 1999) that are closely linked to type 2 diabetes. A recent study reported that SCFAs often derived from gut metabolites suppress fat accumulation (Kimura et al., 2013). As ASP administration attenuated the increase in mWAT and epiWAT weight induced by HFD (Figure 1B), we investigated whether adipose tissue morphology and gene expression patterns were changed by ASP. On histopathologic examination, adipocytes in the mWAT, epiWAT, and BAT were hypertrophic in HFD-fed mice, an effect that was completely prevented by ASP (Figures 4A, 4B, and S3A). The adipocyte diameter was decreased in mWAT, epiWAT, and BAT (Figures 4A, 4B, and S3A). In BAT, transcript levels of *Ucp1,* which uncouples oxidative phosphorylation and utilizes β-oxidation of fatty acids released from triacylglycerol, were remarkably induced in the ASP group. Furthermore, transcript levels of *Dio2* and *Pgc1*α increased by ASP treatment; *Dio2* and *Pgc1*α are two principal players that facilitate the induction of genes involved in BAT thermogenesis. Also, mRNA levels of *Ppar*δd were markedly increased in the ASP group (Figure 4A); the PPAR family of nuclear receptors is the main regulator of fatty acid β-oxidation and energy homeostasis in mitochondria. These data support the fact that ASP induces the miniaturization at the histological level in BAT.

In WAT, mRNA levels of tumor necrosis factor alpha (*Tnf*α) and of monocyte chemoattractant protein 1 (*Mcp1*) that regulate migration and infiltration of monocytes/macrophages were reduced by ASP. Also, the plasma protein level of TNFα and MCP-1 were decreased in accordance with results of relative mRNA expressions (Figure 4C). Furthermore, the mRNA level of the fibrosis marker, collagen type 1 alpha1 (*Col1a1*), was decreased upon ASP treatment. In addition, the transcripts coding for the lipolytic enzyme lipoprotein lipase (*Lpl*) and carnitine palmitoyl transferase 1 (*Cpt1*) were significantly increased after ASP treatment (Figures 4B and S3A). Of note is the fact that transcript levels were altered similarly in both mWAT and epiWAT.

### ASP Improves Intestinal Barrier Function

It has been reported that obesity is accompanied by a compromised intestinal barrier function, which in turn is involved in the development of insulin resistance and impaired glucose tolerance (Monk et al., 2019). In our study of the gut microbial structural community, *Akkermansia* was increased in ASP-supplemented mice. The presence of *Akkermansia* is associated with improved metabolic control and a reduction of inflammation (Anhê et al., 2015). According to microbiome analysis, we investigated intestinal barrier function. In qPCR analysis, transcript levels of the anti-bacterial peptides (*Reg3*γ, *Pla2g2*, *Defa*); of markers of the intestinal mucous layer (*Muc2*, *Muc3*), which protect the epithelium against noxious agents, viruses, and pathogenic bacteria (Hartmann et al., 2016); and of players in tight junctions (*Tjp1*, *Tjp2*) are induced in the ASP supplementation group (Figure 4D). Furthermore, plasma LPS concentration was decreased in ASP-supplemented group (Figure 4E). These findings hence suggest that ASP improves intestinal barrier function, which was corroborated by the tight positive correlation between *Akkermansia* abundance and expression of *Muc2* and *Reg3*γ (Figure 4F).

## Discussion

Our study addressed the mechanism of how ASP, the active ingredient of *Eucommia* leaf, improves metabolic health. Our findings revealed that changes in the gut microbiota community are an important aspect involved in the efficacy of ASP to improve obesity and associated phenotypes, such as insulin resistance and glucose intolerance.

ASP changes the gut microbiota at the phylum level with an important impact on the ratio of Bacteroidetes/Firmicutes. Previous studies suggested that microbiota in obese mice are enriched in Firmicutes and decreased in Bacteroides (Ridaura et al., 2013), an effect that we also observed in our HFD-fed mice. Importantly, ASP administration decreased the amount of Firmicutes and increased that of Bacteroidetes.

Also, at the genus level, ASP induced changes and increased the amounts of *Parabacteroides*. *Parabacteroides*, one of the 18 core members in the human gut microbiota (Falony et al., 2016), plays important functions in modulating host metabolism (Wang et al., 2019). These findings suggest that gut microbiota is an important target for both prevention and therapeutics of obesity and metabolic dysfunctions. A recent study reported that *Parabacteroides* produces succinate, and succinate supplementation in the diet was shown to decrease hyperglycemia in *Ob/Ob* mice via the activation of intestinal gluconeogenesis (IGN) (Wang et al., 2019). Succinate-activated IGN suppressed hepatic glucose production by decreasing the activity of G6Pase and by increasing the glucose-6-phosphate (Glu6P) content in the liver to modulate glucose homeostasis. Our study also confirmed increased succinate levels in the cecal contents of the ASP-treated mice, suggesting that ASP supplementation may modulate glucose homeostasis through *Parabacteroides*-derived succinate.

*Parabacteroides*, *Roseburia,* and *Anaerostipes* produce SCFA and were significantly increased by ASP treatment. SCFAs are important secondary metabolites that activate certain G protein-coupled receptors (GPCRs) and as such improve metabolite signaling (Kimura et al., 2013). We found that the active GLP-1 concentration was higher in the ASP-treated animals than in HFD groups. GLP-1 is an incretin, which stimulates insulin secretion from pancreatic β-cell in a glucose-dependent fashion (Doyle and Egan, 2007), and increasing and stabilizing GLP-1 levels is an important strategy to manage type 2 diabetes (Nauck et al., 2011). The changes in GLP-1 levels induced by ASP can be explained by alterations in the gut microbiota and their subsequent impact on secondary metabolites. Recently, SCFAs were shown to activate the gut receptors, GPR41/43, and as such stimulate L-cells to release GLP-1. We observed that propionate and a precursor of SCFAs, succinate, were increased by ASP treatment in the cecum, consistent with the enrichment of SCFA-producing bacteria. These findings suggest that ASP may favor GLP-1 secretion by the activation of specific GPCR signaling cascades in colonic L-cells by increasing SCFA-producing bacteria. Furthermore, GPR43 activation by SCFA suppresses insulin-mediated fat accumulation (Kimura et al., 2013); this mechanism may mediate the reduction in mWAT and epiWAT hypertrophy in the ASP groups. Our *in vitro* data showing the absence of a direct effect of ASP on GLP-1 release in cultured NCI-H716 L-cells are consistent with such an indirect effect mediated by the increase in SCFA levels (Figures 1H and S1).

In addition, we found that *Akkermansia muciniphila* is robustly increased in ASP-treated mice. In type 2 diabetes, *Akkermansia* have been closely linked to the attenuation of insulin resistance and obesity (Everard et al., 2011), and a recent clinical study showed that *Akkermansia* supplementation improves obesity (Depommier et al., 2019). It has been reported that the increase in *Akkermansia* suppressed intestinal inflammation, strengthens the barrier function of the intestinal epithelium, suppresses the inflow of LPS, and prevents the development of liver steatosis, insulin resistance, and type 2 diabetes (Cani and de Vos, 2017). Our study shows that *Akkermansia* was robustly induced in the HFD-Asp group. Improved metabolic dysfunctions could be explained by the induction of *Akkermansia*. Furthermore, the changes in gene expression observed in the intestine would support such a hypothesis as transcripts of *Muc2*, the predominant secreted mucin from the ileum and colon, were increased (Velcich et al., 2002), and *Muc2* deficiency in mice is associated with disruption of epithelial homeostasis and the development of colon cancer (Hsu et al., 2017). In combination, our results suggest that ASP has a major role on the gut microbiome, not only altering the production of metabolites with signaling and energetic functions but also potentially strengthening gut barrier function.

In conclusion, our study suggests that ASP is an interesting naturally derived compound that through pleiotropic changes in the gut microbiota and an impact on intestinal barrier function and metabolite production can improve obesity and associated metabolic dysfunction.

### Limitations of the Study

In this study, we revealed that ASP changes gut microbiota composition and increases some beneficial bacteria. However, the detailed mechanism of how ASP affects gut microbiota is unknown. Further study is needed to confirm whether ASP acts like prebiotics and has antibacterial activity. In addition, future studies such as fecal transplantation are needed to strengthen our hypothesis that gut microbiota is beneficial for HFD-Asp-fed mice. Our study shows the effect of BAT by ASP. However, the actual energy expenditure and the beneficial effect of ASP under thermoneutrality are still to be determined.

### Resource Availability

#### Lead Contact

Further information and requests for resources should be directed to and will be fulfilled by the Lead Contact, Mitsuhiro Watanabe (mitsuhiro.keio.hsl@gmail.com).

#### Materials Availability

This study did not generate new unique reagents.

#### Data and Code Availability

Microbiome sequencing data have been deposited at the DDBJ Sequence Read Archive (http://trace.ddbj.nig.ac.jp/dra/) under accession number DRA009825.

## Methods

All methods can be found in the accompanying Transparent Methods supplemental file.

## Supplemental Information

Document S1. Transparent Methods, Figures S1–S3, and Table S1
