# Supplementary material for: Asperuloside Improves Obesity and Type 2 Diabetes through Modulation of Gut Microbiota and Metabolic Signaling
Source: iScience. 2020 Sep 2;23(9):101522. doi: 10.1016/j.isci.2020.101522 (PMC7498753; doi:10.1016/j.isci.2020.101522)
Supplement: Document S1. Transparent Methods, Figures S1–S3, and Table S1 [file mmc1.pdf]

iScience, Volume *23*

Supplemental Information

Asperuloside Improves Obesity and Type 2 Diabetes through Modulation of Gut Microbiota and Metabolic Signaling

Anna Nakamura, Yoko Yokoyama, Kazuki Tanaka, Giorgia Benegiamo, Akiyoshi Hirayama, Qi Zhu, Naho Kitamura, Taichi Sugizaki, Kohkichi Morimoto, Hiroshi Itoh, Shinji Fukuda, Johan Auwerx, Kazuo Tsubota, and Mitsuhiro Watanabe

Transparent Methods

Materials

Asperuloside (ASP) was obtained from KOBAYASHI Pharmaceutical Co., Ltd. Japan.

Animal Studies

All animal experiments were performed in accordance with the standards set forth in the Guidelines for the Use and Care of Laboratory Animals at Keio University, Japan. The protocols were approved by the Institute for Experimental Animals of Keio University. Male C57BL/6J mice of 5 weeks of age, were obtained from Japan SLC, Inc. All mice were maintained in a temperature-controlled (23°C) facility with a 12-h light/dark cycle and were given free access to food and water. The mice were placed on the test diet after one-week acclimation. The control diet and high fat diet were obtained from Research Diets, Inc. The control diet (D12450B) contained 20kcal% protein, 70kcal% carbohydrate, and 10kcal% fat, and the high fat diet (D12492) contained 20kcal% protein, 20kcal% carbohydrate, and 60kcal% fat. For treatment with ASP, mice were fed high fat diets with 0.25% (w/w) ASP. The mice were fasted 6-h before harvesting blood for subsequent blood measurements and tissues for RNA isolation and histology.

mRNA Expression Analysis by Quantitative RT-PCR

Total RNA was extracted from tissue samples using the RNeasy Mini Kit (QIAGEN, Hilden, Germany). The cDNA was synthesized from total RNA with the Prime Script RT Reagent Kit (Takara Bio Inc., Shiga, Japan). The expression levels were analyzed in cDNA synthesized from total mRNA using real time PCR. The sequences of the primer sets used are displayed in Supplemental Table 1.

Oral glucose tolerance test (OGTT) and intraperitoneal insulin tolerance test (IPITT)

The OGTT was performed in animals that were fasted 6-h. Glucose (Otsuka pharmaceutical factory Inc., Tokushima, Japan) was administered by gavage at a dose of 2 g/kg. An IPITT was done in 6-h fasted animals. Insulin (Humalin N, Eli Lilly Japan, Kobe, Japan) was injected at a dose of 0.75 units/kg. Glucose quantification was done with the GUNZE Life check (GUNZE Limited, Osaka, Japan).

Plasma measurements

Lipid composition, plasma total cholesterol and NEFA were determined by enzymatic assay kits from Labo Assay TM series (FUJIFILM Wako Pure Chemical Corporation, Osaka, Japan). TG were measured by determiner L TG-Ⅱ (Hitachi Chemical Diagnostics System Co., Ltd., Chuo-ku, Tokyo). TNFα was quantified by Quantikine ELISA Kit (R&D Systems, MN, USA). MCP-1 was measured by MCP-1 ELIZA kit (Thermo Fisher Scientific, MA, USA). The level of the plasma lipopolysaccharide (LPS) was determined by ELISA Kit for Lipopolysaccharide (LPS) (Cloud-Clone Corp., TX, USA) according to the operating instructions.

Metabolite Extraction and CE-TOFMS-based Metabolome Analysis

Cecal contents were used for metabolite extraction as described previously with slight modification (Ishii et al., 2018). Briefly, 10 mg of freeze-dried cecal contents were suspended in 500μL of methanol supplemented with the internal standards (20μM each of methionine sulfone and D-camphor-10-sulfonic acid (CSA)). The mixture was shaken vigorously with three 3-mm beads and 100mg of 0.1-mm zirconia beads (BioSpec Products, Inc., OK, USA) by ShakeMaster® NEO (Biomedical Science, Tokyo, Japan). After extraction of cecal metabolites by standard chloroform-methanol extraction method, the aqueous phase was centrifuged at 4600 × g for 15min at 20°C, and the supernatant was transferred to a 5-kDa-cutoff filter column (Ultrafree MC-PHHCC 250 / pk for Metabolome Analysis, (Human Metabolome Technologies, Tsuruoka, Japan) and centrifuged at 9100 × g for 3 - 7h at 4°C. The filtrate sample was dried under vacuum and the residue then was dissolved in 40 μ of Milli-Q water containing reference compounds (200 μ M each of 3-aminopyrrolidine and time state). The level of extracted metabolites were measured in both positive and negative modes using CE-TOFMS as described previously (Sugimoto et al., 2010). All CE-TOFMS experiments were performed using an Agilent capillary electrophoresis system (Agilent Technologies, CA, USA). Annotation tables were produced from measurement of standard compounds and were aligned with the datasets according to similar m/z value and normalized migration time. Then, peak areas were normalized against those of the internal standards methionine sulfone and CSA for cationic and anionic metabolites, respectively. Concentrations of each metabolite were calculated based on their relative peak areas and concentrations of standard compounds. The statistical analysis was performed using online tool Metaboanalyst (Xia and Wishart, 2011).

DNA Extraction

DNA from cecal contents was extracted following methods as described previously (Furusawa et al., 2013). In brief, cecal content samples were initially freeze dried by using VD-800R lyophilizer (TAITEC, Saitama, Japan) for at least 12 hours. Samples were disrupted with 3-mm Zirconia Beads by vigorous shaking (1,500rpm, for 10 min) using Shake Master (Biomedical Science, Tokyo, Japan). About 10 mg cecal contents were suspended with DNA extraction buffer containing 200 μL of 10% (w/v) SDS/TE (10 mM Tris-HCl, 1 mM EDTA, and pH 8.0) solution, 400 μL of phenol/chloroform/isoamyl alcohol (25 : 24 : 1), and 200 μL of 3 M sodium acetate. Obtained emulsions were further disrupted with 0.1 mm zirconia/silica beads by vigorous shaking (1,500 rpm. for 5 min) using Shake Master. After centrifugation at 17,800 ×g for 5 min at 20°C, bacterial genomic DNA was purified by the standard phenol/chloroform/isoamyl alcohol protocol. RNAs in the sample were removed by RNase A treatment, and then DNA samples were purified again by the standard phenol/chloroform/isoamyl alcohol treatment.

Microbiome analysis by 16S rRNA gene sequencing

Genomic DNAs extraction was performed previously with some modifications (Ishii et al., 2018). 16S rRNA genes in the fecal DNA samples were analyzed using a MiSeq sequencer (Illumina, CA, USA). The V1-V2 region of the 16S rRNA genes was amplified from the DNA isolated from cecal contents using bacterial universal primer set 27Fmod (5′ -AGRGTTTGATYMTGGCTCAG-3′) and 338R (5′ -TGCTGCCTCCCGTAGGAGT-3′). PCR was performed with Tks Gflex DNA Polymerase (Takara Bio Inc., Shiga, Japan) and amplified according the following program: one denaturation step at 98°C for 1 min, followed by 20 cycles of 98°C for 10 s, 55°C for 15 s, and 68°C for 30 s, and a final extension step at 68°C for 3 min. The amplified products were purified using Agencourt AMPure XP (Beckman Coulter) and then further amplified using forward primer (5′ -AATGATACGGCGACCACCGAGATCTACAC- NNN NNNNN-TATGGTAATTGT-AGRGTTTGATYMTGGCTCAG-3′) containing the P5 sequence, a unique 8 bp barcode sequence for each sample (indicated in N), Rd1 SP sequence and 27Fmod primer and reverse primer (5′ -CAAGCAGAAGACGGCATACGAGAT-NNNNNNNN-AGTCAGTCAGCC- TGC TGCCTCCCGAGGAGT-3′) containing the P7 sequence, a unique 8-bp barcode sequence for each sample (indicated by strings of Ns), Rd2 SP sequence, and 338R primer. After purification using Agencourt AMPure XP, mixed sample was prepared by pooling approximately equal amounts of PCR amplicons from each sample. Finally, MiSeq sequencing was performed according to the manufacturer's instructions. In this study, 2 × 300 bp paired-end sequencing was employed.

Microbiome Data Analysis Using QIIME

First, to assemble the paired end reads, fast length adjustment of short reads (FLASH) (v1.2.11) (Magoc and Salzberg, 2011) was used. Assembled reads with an average -value < 25 were filtered out using in-house script. 5,000 filter-passed reads were randomly selected from each sample and used for further analysis. Reads were then processed using quantitative insights into microbial ecology (QIIME) (v1.8.0) pipeline (Caporaso et al., 2010). Sequences were clustered into operational taxonomic units (OTUs) using 97% sequence similarity and OTUs were assigned to taxonomy using RDP classifier.

Sequence accession number

The microbiome analysis data have been deposited at the DDBJ Sequence Read Archive (http://trace.ddbj.nig.ac.jp/dra/) under accession number DRA009825.

Histology and quantification of the adipocyte.

Adipose tissues were harvested and immediately fixed Tissue-Tek UFIX (Sakura Finetek Japan, Tokyo, Japan). Hematoxylin and eosin (H&E) staining was conducted using paraffin-embedded tissue sections. The adipocyte diameter was quantified by using ImageJ software (NIH, MD, USA) (Schneider et al. 2012).

Cell culture

In vitro experiments were performed with NCI-H716 cells in RPMI1640 medium with 10% FBS with vehicle (DMSO), TCA, or ASP at the indicated concentrations. Measurement of GLP-1 release was measured in cultural medium after treatments with the chemical compounds, with supernatants of the medium containing a protease inhibitor cocktail (Roche Diagnostics K.K., Tokyo, Japan). GLP-1 concentration was measured using the GLP-1 Active Assay Kit from IBL (IBL Co., Gunma, Japan). The GLP-1 concentrations were normalized against total cell amounts measured by TaKaRa BCA protein Assay Kit (Takara Bio Inc., Shiga, Japan).

Statistical analysis

Values were reported as mean ± SEM. Statistical differences were determined by either one-way ANOVA or Student's t test with using Graph Pad Prism 8. Statistical significance is displayed as follows: *p < 0.05, **p < 0.01, ***p < 0.001 HFD vs HFD-Asp, #p < 0.05, ##p < 0.01, ###p < 0.001 HFD vs Control, +p < 0.05, ++p < 0.01, +++p < 0.001 Control vs HFD-Asp.

References

Caporaso, J.G., Kuczynski, J., Stombaugh, J., Bittinger, K., Bushman, F.D., Costello, E.K., Fierer, N., Peña, A.G., Goodrich, J.K., Gordon, J.I., et al. (2010). QIIME allows analysis of high-throughput community sequencing data. Nat. Methods 7, 335–336.

Ishii, C., Nakanishi, Y., Murakami, S., Nozu, R., Ueno, M., Hioki, K., Aw, W., Hirayama, A., Soga, T., Ito, M., et al. (2018). A Metabologenomic Approach Reveals Changes in the Intestinal Environment of Mice Fed on American Diet. Int. J. Mol. Sci. 19, 4079.

Sugimoto, M., Wong, D.T., Hirayama, A., Soga, T., and Tomita, M. (2010). Capillary electrophoresis mass spectrometry-based saliva metabolomics identified oral, breast and pancreatic cancer-specific profiles. Metabolomics 6, 78–95.

Schneider, C.A., Rasband, W.S., Eliceiri, K.W. (2012). NIH Image to ImageJ: 25 years of image analysis. Nat. Methods 9, 671-675.

Xia, J., and Wishart, D.S. (2011). Web-based inference of biological patterns, functions and pathways from metabolomic data using MetaboAnalyst. Nat. Protoc. 6, 743–760.

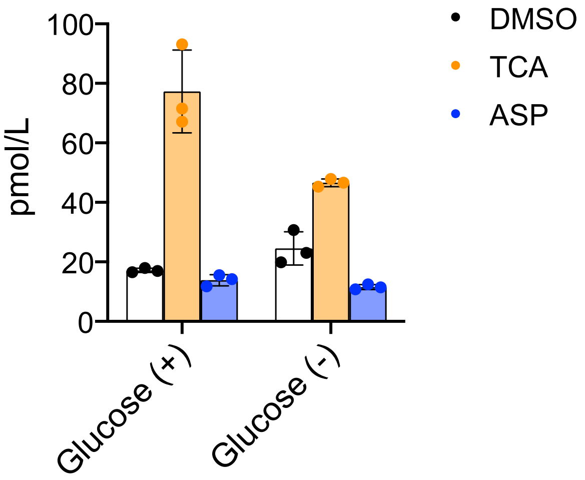

Figure S1. Related to Figure 1. Asperuloside (ASP) stimulates GLP-1 secretion indirectly. GLP-1 concentration secreted from NCI-H716 cell line cultured for 30 minutes in the absence or presence of glucose and either treated with DMSO (vehicle), taurocholic acid (100µM), or ASP (100µM) added within the medium.

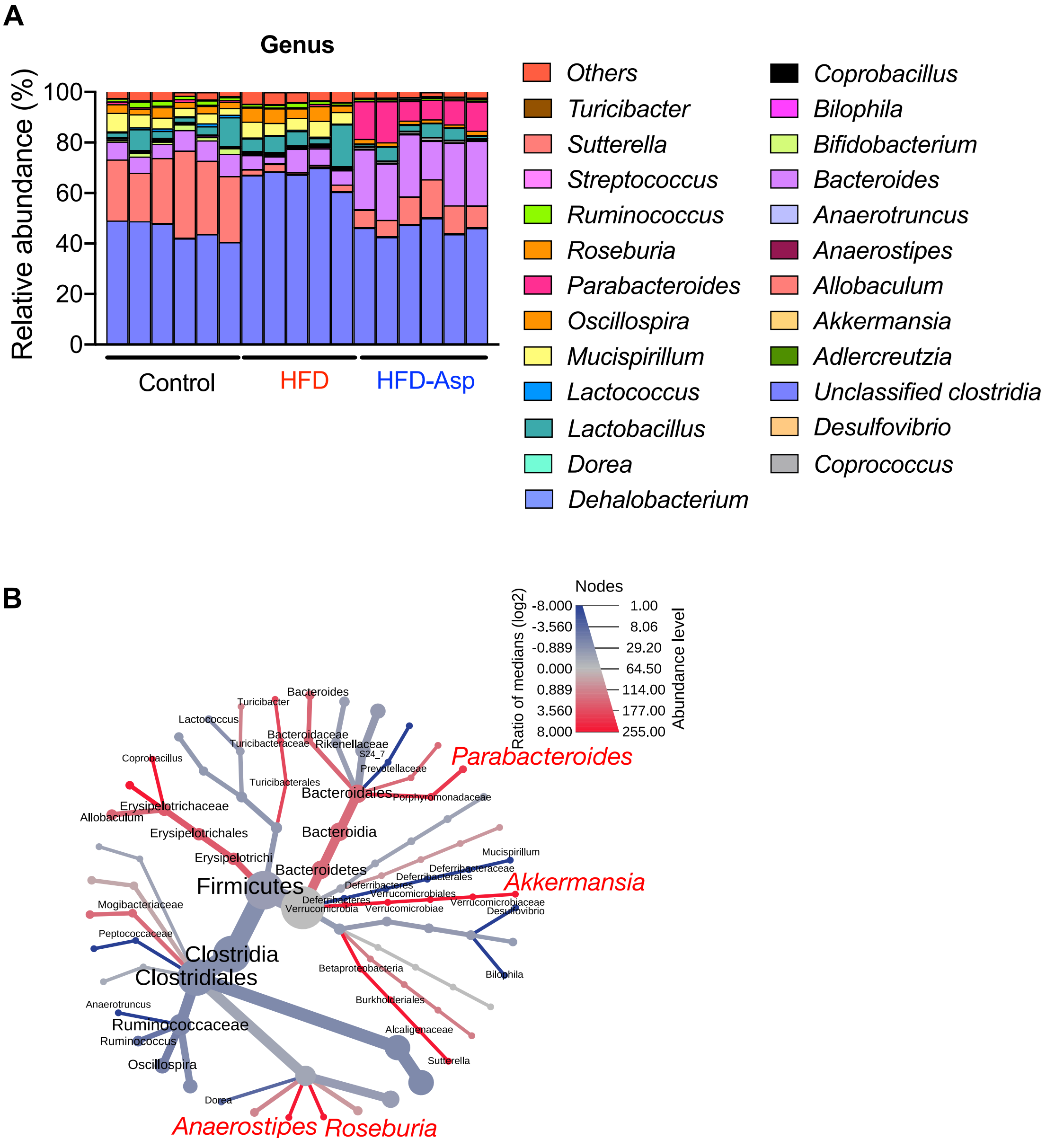

Figure S2. Related to Figure 2. ASP changes gut microbiota community and increases specific bacteria in genus level. Analysis of 16s rRNA gene sequences on the mice used in Figure 1. (A) Genus level taxonomic distributions of the microbial communities in cecal contents of mice fed with the 3 different diets. (B) Bacterial community structure at genus level represented with a heat tree compared high-fat diet (HFD) fed mice and HFD +0.25% asperuloside fed mice.

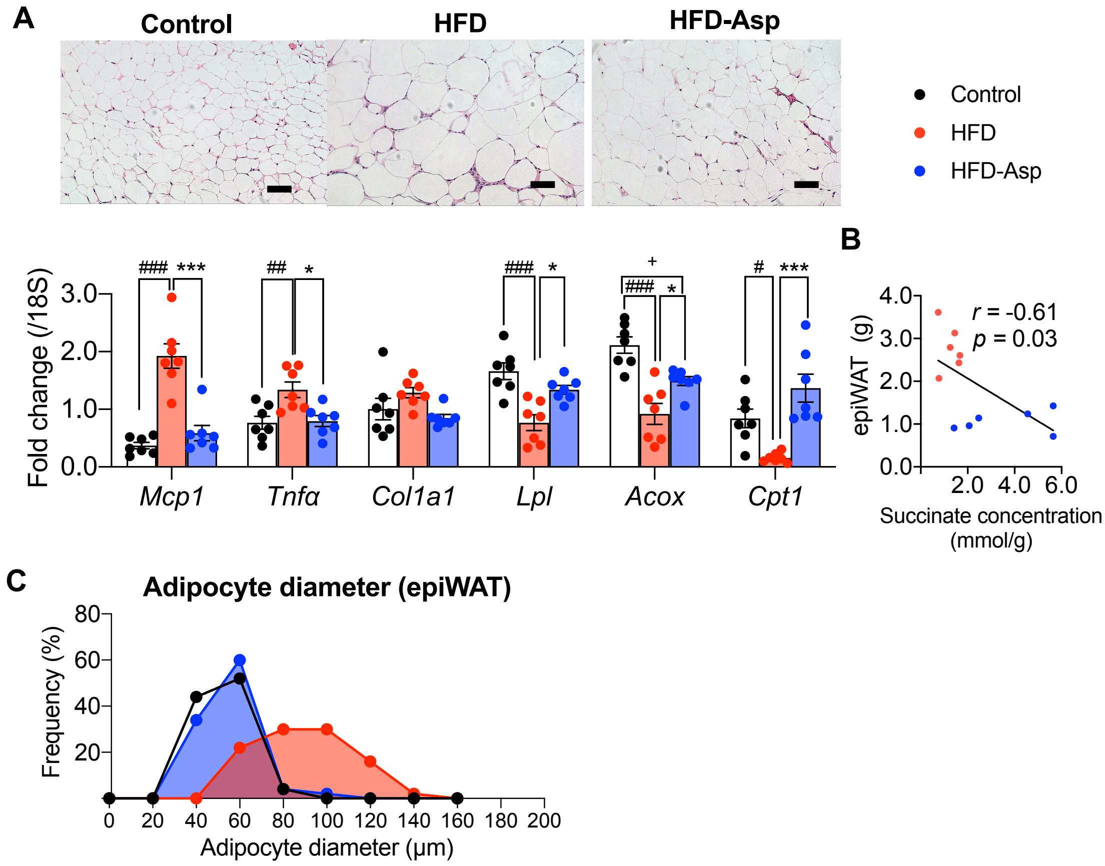

Figure S3. Related to Figure 4. ASP changes adipocyte metabolism. Analysis on the mice used in Figure 1. (A) Histological analysis and expression of mRNA levels of selected genes qPCR analysis in the epididymal white adipose tissue (epiWAT). Adipocyte morphology was assessed by H&E staining (Scale bar: 50μm). (B) Correlation analysis between succinate concentration and epiWAT weight. Y axis: weight of epiWAT, X axis: succinate concentration of cecal contents. (C) Adipocyte diameter. Results are expressed as mean ± SEM (n = 6-7 mice for each group). *p < 0.05, **p < 0.01, ***p < 0.001 HFD vs HFD-Asp, #p < 0.05, ##p < 0.01, ###p < 0.001 HFD vs Control, +p < 0.05, ++p < 0.01, +++p < 0.001 Control vs HFD-Asp. Statistical analysis with one-way ANOVA followed Tukey's multiple comparison test.

Table S1. Related to Figure 4. Primer sequences.

Gene	Forward primer (5'→3')	Reverse primer (5'→3')
18S	TTCTGGCCAACGGTCTAGACAAC	CCAGTGGTCTTGGTGTGCTGA
Acox	TTCTACCAATCTGGCTGCAC	GTGGGTGGTATGGTGTCGTA
Col1a1	CAACCTGGACGCCATCAAG	CGTGGAATCTTCCGGCTGTAG
Cpt1	TCATTGGCCACCAGTTCCATTA	CCAATGGCTGCCACACTCTC
Dio2	TTCTGAGCCGCTCCAAGT	GGAGCATCTTCACCCAGTTT
Defa	GGTGATCATCAGACCCCAGCATCAGT	AAGAGACTAAAACTGAGGAGCAGC
Lpl	CAGCAGGGAGTCAATGAAGA	ATCCGTGTGATTGCAGAGAG
Mcp1	CTGGATCGGAACCAAATGAG	CGGGTCAACTTCACATTCAA
Muc2	GGGAGGGTGGAAGTGGCATTGT	TGCTGGGGTTTTTGTGAATCTC
Muc3	AACTGCAGCTACGGCAAATGTC	AGGTTTCGCCTACCATCGTAAC
Pgc1α	AAGGGCCAAACAGAGAGAGA	GCGTTGTGTCAGGTCTGATT
Pla2g2	AGGATTCCCCAAGGATGCCAC	CAGCCGTTTCTGACAGGAGTTCTGG
Pparδ	TCTGCCATCTTCTGCAGCAGCTT	CTCTTCATCGCGGCCATCATTCT
Prdm16	CAGCACGGTGAAGCCATTC	GCGTGCATCCGCTTGTG
Reg3γ	CTGGGACAGTGACCTGGACT	GCACCTCAGGGAAGAGTCTG
Tnfα	CTGGGACAGTGACCTGGACT	GCACCTCAGGGAAGAGTCTG
Tjp1	GACCAATAGCTGATGTTGCCAGAG	TATGAAGGCGAATGATGCCAGA
Tjp2	GACATCTATGCGGTTCCAATCAA	TGGTGTCCTGGTAAAGTCTGGAAG
Ucp1	GGCCCTTGTAAACAACAAAATAC	GGCAACAAGAGCTGACAGTAAAT
Ucp2	AGAAGTGAAGTGGCAAGGGA	GCTGAGCTGGTGACCTATGA
